# Endogenous 17β-estradiol is required for activity-dependent long-term potentiation in the striatum: interaction with the dopaminergic system

**DOI:** 10.3389/fncel.2015.00192

**Published:** 2015-05-27

**Authors:** Alessandro Tozzi, Antonio de Iure, Michela Tantucci, Valentina Durante, Ana Quiroga-Varela, Carmela Giampà, Michela Di Mauro, Petra Mazzocchetti, Cinzia Costa, Massimiliano Di Filippo, Silvarosa Grassi, Vito Enrico Pettorossi, Paolo Calabresi

**Affiliations:** ^1^Department of Experimental Medicine, Section of Physiology and Biochemistry, University of PerugiaPerugia, Italy; ^2^Fondazione Santa Lucia, IRCCSRome, Italy; ^3^Clinica Neurologica, Dipartimento di Medicina, Università degli Studi di Perugia, Ospedale Santa Maria della MisericordiaPerugia, Italy

**Keywords:** estrogen receptors, P450-aromatase, striatum, synaptic plasticity, long-term potentiation, medium spiny neurons, cholinergic interneurons, D1 receptor

## Abstract

17β-estradiol (E2), a neurosteroid synthesized by P450-aromatase (ARO), modulates various brain functions. We characterized the role of the locally synthesized E2 on striatal long-term synaptic plasticity and explored possible interactions between E2 receptors (ERs) and dopamine (DA) receptors in the dorsal striatum of adult male rats. Inhibition of E2 synthesis or antagonism of ERs prevented the induction of long-term potentiation (LTP) in both medium spiny neurons (MSNs) and cholinergic interneurons (ChIs). Activation of a D1-like DA receptor/cAMP/PKA-dependent pathway restored LTP. In MSNs exogenous E2 reversed the effect of ARO inhibition. Also antagonism of M1 muscarinic receptors prevented the D1-like receptor-mediated restoration of LTP confirming a role for ChIs in controlling the E2-mediated LTP of MSNs. A novel striatal interaction, occurring between ERs and D1-like receptors in both MSNs and ChIs, might be critical to regulate basal ganglia physiology and to compensate synaptic alterations in Parkinson’s disease.

## Introduction

Estrogens, in particular 17β-estradiol (E2), play a fundamental role in regulating brain activity modulating neuronal expression of enzymes, receptors, structural proteins and synaptic plasticity and notably influencing cognition and behavior (Wong and Moss, 1992; Murphy and Segal, 1996; McEwen, 2002; Kramár et al., 2013). The E2 effects depend on genomic responses *via* nuclear receptors (Paech et al., 1997) and rapid non-genomic responses involving membrane receptors (ERα/β and GPER-1) (Kelly et al., 1976, 2002; Wong and Moss, 1992; Kelly and Levin, 2001; Qiu et al., 2003; Toran-Allerand, 2004; Revankar et al., 2005; Pedram et al., 2006; Morissette et al., 2008; Raz et al., 2008; Boulware and Mermelstein, 2009; Almey et al., 2012). The ERs may be also activated by extracellular agents initiating intracellular transduction pathways and transcriptional activity in the absence of E2. For example, ERs are activated in a ligand-independent manner by dopamine (DA; Power et al., 1991; Olesen et al., 2005) and by the D1-like receptor (D1R) agonist SKF-82958, stimulating ER-dependent activation of intracellular signaling pathways (Walters et al., 2002) and inducing lordosis in rats primed with E2 (Apostolakis et al., 1996). Moreover, in the nucleus striatum E2 activates membrane-localized ERs to modulate DA synaptic neurotransmission (Becker, 1990a), calcium channel activity (Mermelstein et al., 1996) and motor activity (Hampson and Kimura, 1988; Becker, 1990b). This is consistent with the observation that E2 rapidly enhances amphetamine-induced rotational behavior (Becker, 1990a). Moreover, E2 can activate metabotropic glutamate receptor signaling, affecting cAMP-response-element-binding protein (CREB) that plays an important role in activity-dependent neuronal plasticity (Boulware et al., 2005; Boulware and Mermelstein, 2009). Therefore, the interplay between DA and E2 is likely to occur for modulating neuronal activity and plasticity in striatal neurons. However, it is unknown whether DA interacts with the circulating E2 or with the E2 that can be locally synthesized in the nervous system from testosterone through P450-aromatase (ARO) (Naftolin et al., 1975; Simpson et al., 1994; Balthazart et al., 2001; Kimoto et al., 2001; Hojo et al., 2004, 2008; Balthazart and Ball, 2006; Mukai et al., 2006). Recently, it has been shown a decisive role of endogenous E2 in inducing long-term potentiation (LTP) in the vestibular nuclei by acute effects (Grassi et al., 2009, 2010; Scarduzio et al., 2013) and in the hippocampus by both acute (Grassi et al., 2011; Tanaka and Sokabe, 2012; Pettorossi et al., 2013) and chronic influences (Vierk et al., 2012, 2014). Based on this evidence, considering the presence of ARO and ERs in the nucleus striatum (Küppers and Beyer, 1998, 1999; Almey et al., 2012), we hypothesized a role for endogenous E2 also in the plasticity of striatal neurons. Moreover, since DA is critical for striatal LTP (Calabresi et al., 2007) we assumed a key interaction between E2 and DA in this event. Thus, in the present study we investigated the involvement of endogenous E2 in the expression of striatal synaptic plasticity and its interaction with DA demonstrating a critical cross-talk between E2 and D1-like DA-dependent signaling.

## Materials and Methods

### Ethic Statement on Animal Use

All procedures were conducted in conformity with the European Communities Council Directive of November 1986 (86/609/ECC), in accordance with protocols approved by the Animal Care and Use Committee at the Universities of Perugia (Italy). Wistar rats (Harlan) (2 per cage) were kept under regular lighting conditions (12 h light/dark cycle) and given food and water *ad libitum*. All efforts were made to minimize the number of animals used and their suffering.

### Electrophysiology

Two- to three-month-old male rats were sacrificed, under deep halothane anesthesia, by cervical dislocation. The brain was rapidly removed and coronal corticostriatal slices (250–270 μm) were cut in Krebs’ solution (in mM: 126 NaCl, 2.5 KCl, 1.2 MgCl_2_, 1.2 NaH_2_PO_4_, 2.4 CaCl_2_, 10 glucose, 25 NaHCO_3_) using a vibratome. The slices were maintained in Kreb’s solution, bubbled with a O_2_ 95% and CO_2_ 5% gas mixture at room temperature (Calabresi et al., 1992). A single coronal slice including the cortex and the striatum was transferred to a recording chamber and submerged in a continuously flowing Kreb’s solution (34°C; 2.5–3 ml/min) bubbled with a 95% O_2_–5% CO_2_ gas mixture. Intracellular sharp microelectrode recordings were performed in most of the experiments. In addition, whole-cell voltage-clamp recordings were used for LTP recordings of ChIs and to explore MSN intracellular pathways. Only neurons electrophysiologically identified as MSNs or ChIs were considered for experiments (Calabresi et al., 1992). For intracellular recordings, sharp microelectrodes were filled with 2 mM KCl and an Axoclamp 2B amplifier (Molecular Devices) was used. For patch-clamp recordings, neurons were visualized using differential interference contrast (Nomarski) and infrared microscopy (Olympus). Whole-cell voltage-clamp recordings (Vhold −70 mV) were performed with borosilicate glass pipettes (4–7 MΩ; Ra 15–30 MΩ) filled with a standard internal solution containing (in mM): 125 K^+^-gluconate, 0.1 CaCl_2_, 2 MgCl_2_, 0.1 EGTA, 10 HEPES, adjusted to pH 7.3 with KOH. Signals were amplified with a Multiclamp 700B amplifier (Molecular Devices), recorded and stored on PC using pClamp 10 (Molecular Devices). In all patch-clamp experiments 50 μM picrotoxin was added to the external medium to block GABA_A_ receptors. The recording electrodes were placed within the dorsolateral striatum. Glutamatergic excitatory postsynaptic potentials (EPSPs) and currents (EPSCs) were evoked every 10 s by means of a bipolar electrode connected to a stimulation unit (Grass Telefactor) and located in the white matter between the cortex and the striatum to activate glutamatergic fibers.

To induce LTP or LTD we used a high-frequency stimulation (HFS) protocol consisting of three trains of 3 s (20 s interval) at 100 Hz. During the HFS protocol, the stimulus intensity (10–20 V; 30–40 μs) was increased to supra-threshold levels. The same stimulation protocol was used to induce LTP in ChIs. External Mg^2+^ ions were omitted to maximize the contribution of NMDA receptors during LTP experiments in MSNs (Calabresi et al., 1992). To induce depotentiation of previous obtained LTP we used 15 min of low frequency stimulation (LFS) at 1 Hz delivered at the same stimulus intensity.

### Rats with 6-Hydroxydopamine-Induced Lesion

Three months old 6-hydroxydopamine (6-OHDA)-lesioned male rats were obtained as previously reported (Picconi et al., 2003). Briefly, rats were deeply anesthetized with chloral hydrate (400 mg/ml/Kg) and injected unilaterally with 6-OHDA (12 μg/4 μl of saline containing 0.1% ascorbic acid) *via* a Hamilton syringe into the medial forebrain bundle at a rate of 0.38 μl/min (AP −4.4, ML +1.2, DL −7.8). Fifteen days after surgery rats were tested with 0.05 mg/Kg subcutaneous apomorphine and rotations, contra-lateral to the lesioned side of the brain, were counted for 40 min. Only those rats consistently making at least 200 contra-lateral turns were enrolled in the study. The severity of the lesion was also quantified afterward by nigral TH immunohistochemistry. Sham-operated rats were injected only with saline. Experiments were performed 4–6 weeks after apomorphine injection (Picconi et al., 2003).

### Drugs

Drugs were applied by dissolving them to the desired final concentration in the Kreb’s solution and by switching the perfusion from control solution to drug-containing solution. 8-Br-cAMP, Dopamine (DA), 17-β-Estradiol (E2), Letrozole (LET), ICI-182780 (ICI), Picrotoxin, PD98059, Pirenzepine, Quinpirole, RP-cAMPS, SCH 23390, SKF-38393 (SKF), were purchased from Tocris-Cookson (Bristol, UK). Drugs applied in the recording chamber were delivered for at least 10 min before induction of long-term synaptic effects and maintained throughout the experiment. In some patch-clamp experiments 8-Br-cAMP or RP-cAMPS was added to the internal solution.

### Statistical Analysis

Data analysis was performed off-line using Clampfit 10 (Molecular Devices) and GraphPad Prism 5 (GraphPad Software). Values given in the text and figures are mean ± S.E., *n* representing the number of recorded neurons. Only one neuron per slice was recorded. Changes of EPSP or EPSC amplitude induced by drugs or by stimulation protocols were expressed as a percentage of the baseline, the latter representing the normalized EPSP or EPSC mean amplitude acquired during a stable period (10–15 min) before delivering drugs or stimulation. LTP or LTD presence was statistically verified (Student’s paired *t-*test) by comparing in each experiment the value of the EPSP or EPSC amplitude at 25–30 min after the application of the stimulating protocol with the baseline. For the analysis of the depotentiation we compared the EPSP amplitudes immediately before LFS (5 min) and the amplitude at 20 min after the LFS induction (5 min). In addition, the possible modifications of the baseline or the long-term effects induced by drugs were statistically verified by comparing values 5 min before with those at 10–15 min post-drug (Student’s paired *t* test). The magnitude of long-term effects induced in different experimental conditions were compared using the two-way analysis of variance (ANOVA). The significance level was established at *P* < 0.05 for Student’s *t* test and ANOVA.

## Results

### Effect of the Inhibition of E2 Synthesis or E2 Receptors Antagonism on MSNs Basal Membrane Properties

**Figure 1:**
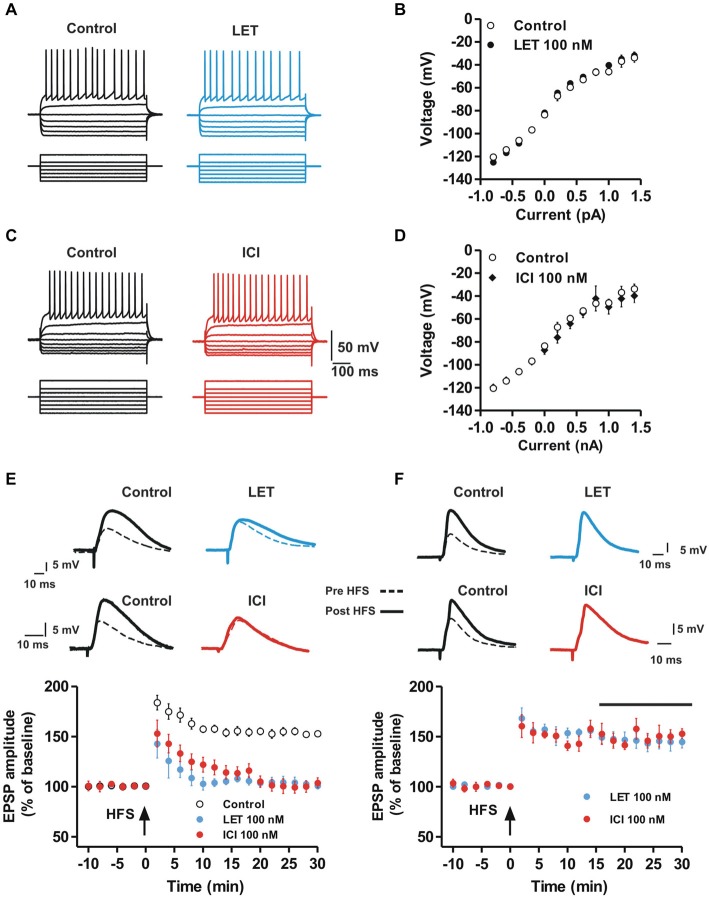
**Aromatase inhibition and ER antagonism prevent the induction but not the maintenance of MSN LTP while basal membrane properties are unaffected. (A,C)** Voltage traces recorded from MSNs during the injection of hyperpolarizing and depolarizing steps of currents in control conditions (left) and in the presence of LET (**A**, right) or ICI (**C**, right). **(B,D)** Voltage-current plots of MSNs membrane potentials acquired in control conditions (white circles) and in the presence of LET (**B**, black circles) and ICI (**D**, black diamonds). **(E)** EPSP pairs of traces recorded from striatal MSNs before and after the application of a HFS protocol in control conditions (left traces) and in the presence of 100 nM letrozole (LET, right traces, top) or 100 nM ICI-182,780 (ICI, right traces, bottom). The time-courses show the effect of HFS (arrow) applied in control condition (white circles) and in the presence of LET (black circles) or ICI (grey circles) on the EPSP amplitude. **(F)** EPSP pairs recorded before and after HFS application in control conditions (left) and in the presence of LET (top) or ICI (bottom) applied 15 minutes after the HFS (black bar). The time-courses show the effect of LET or ICI on the maintenance of HFS-LTP. Note that LTP induction, but not the maintenance, is prevented in the presence of LET or ICI.

In order to analyze possible effects of endogenous E2 and ER activation on the basal membrane properties of the MSNs, we evaluated the firing pattern discharge and current-voltage relationship applying hyperpolarizing and depolarizing steps of currents to MSNs in control condition and in the presence of letrozole (LET) (Figures 1A,B) or ICI-182780 (ICI) (Figures 1C,D). Both LET and ICI did not change the current-voltage relationship of the recorded neurons (Control, *n* = 9 vs. LET, *n* = 10, ANOVA, *P* > 0.05; Control, *n* = 9 vs. ICI, *n* = 9, ANOVA, *P* > 0.05), suggesting that the local synthesis of E2, as well as ER activation, do not affect the basal membrane properties of MSNs. Furthermore, the application of LET or ICI (*n* = 6 for each group) to the slices did not also alter *per se* the amplitude of the EPSP (Student’s *t* test, *P* > 0.05, data not shown). The lack of effect on the membrane electrical properties and on synaptic response excludes a basal activity of ARO and a tonic effect of endogenous E2 on neuronal excitability and synaptic responsiveness.

### Inhibition of Striatal E2 Synthesis and E2 Receptor Antagonism Prevent the Induction of LTP but not LTD or Depotentiation in Striatal MSNs

We subsequently analyzed a possible role of endogenous E2 and ER activation in the induction of LTP, LTD and depotentiation of glutamatergic synaptic transmission in MSNs. EPSPs were recorded before and after the application of the HFS protocol to induce LTP (Calabresi et al., 1992). In control condition the EPSP amplitude was increased by 53 ± 2.4% (*n* = 10) following the HFS, while in the presence of 100 nM of the ARO inhibitor LET or 100 nM of the ER antagonist ICI, the induction of LTP was completely prevented in all the recorded MSNs (LET, *n* = 9; ICI, *n* = 10; Figure 1E). Conversely, the maintenance of LTP was unaffected by both LET and ICI. In fact, drugs administered 15 min after the LTP induction did not significantly modify the amplitude of the EPSP (LET, *n* = 5, ICI, *n* = 5; Figure 1F) suggesting a pivotal role of endogenous E2 in the induction phase, but not in the maintenance, of MSN LTP.

**Figure 2:**
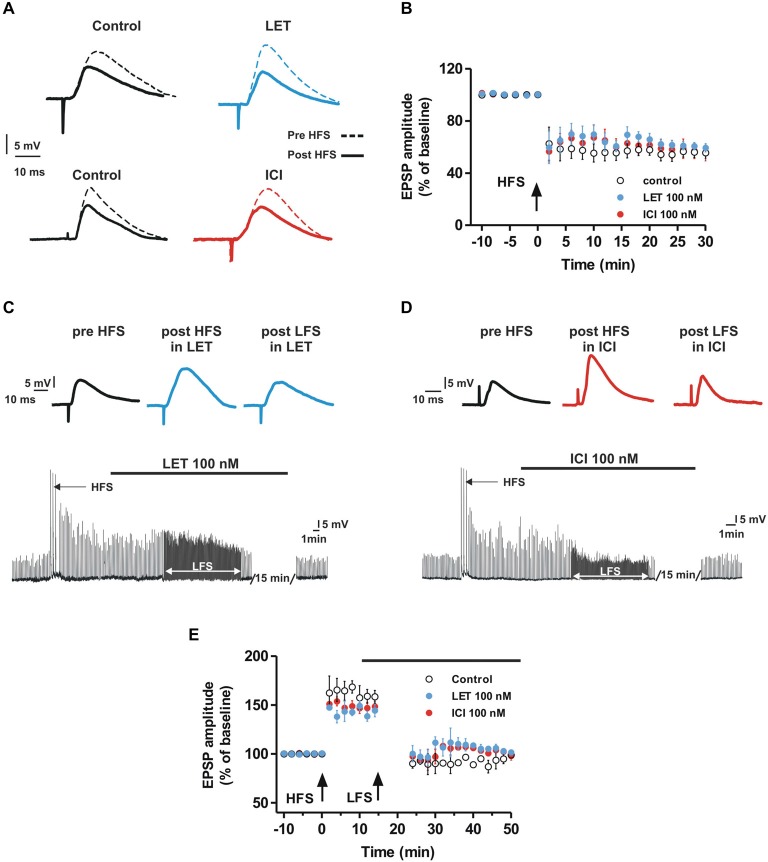
**Aromatase inhibition and ER antagonism do not affect LTD and depotentiation in the MSNs. (A,B)** EPSP pairs **(A)** and time-courses of the EPSP amplitude **(B)** recorded from MSNs before and after the HFS application showing the LTD induction in control conditions and in the presence of LET or ICI). **(C,D)** EPSP traces of a MSN recorded before the HFS, after the HFS in the presence of LET **(C)** or ICI **(D)** and after the LFS in the presence of LET **(C)** or ICI **(D). (E)** Time-courses of MSN EPSP amplitudes acquired in control conditions and in the presence of LET or ICI (black bar) showing no differences in the depotentiation between the groups of MSNs.

We also explored the possible role of E2 synthesis and ER activation on the induction of LTD in MSNs. In control conditions, the LFS protocol induced LTD of synaptic transmission, in fact the EPSP amplitude was reduced by 44.4 ± 4.4% (*n* = 6). LTD was also induced in the presence of LET (40.6 ± 3.2%, *n* = 6) or ICI (44.7 ± 6.1%, *n* = 6) showing an amplitude that was not significantly different from control (Control vs. LET, ANOVA, *F*_(20,210)_ = 0.39, *P* = 0.99; Control vs. ICI, ANOVA, *F*_(20,210)_ = 0.23, *P* = 1.00; Figures 2A,B). We also analyzed the possibility that E2 affected the depotentiation, a form of synaptic plasticity that can be observed in MSNs after applying an LFS protocol following the LTP induction (Picconi et al., 2003). As shown in Figures 2C–E, HFS produced a robust LTP (*n* = 12) that was cancelled by LFS both in control condition (*n* = 4) and in the presence of LET (*n* = 4) or ICI (*n* = 4) applied 10 min after the induction of LTP.

### E2 Application Restores LTP of MSNs in the Presence of ARO Inhibition but not in the Presence of ER Antagonism

**Figure 3:**
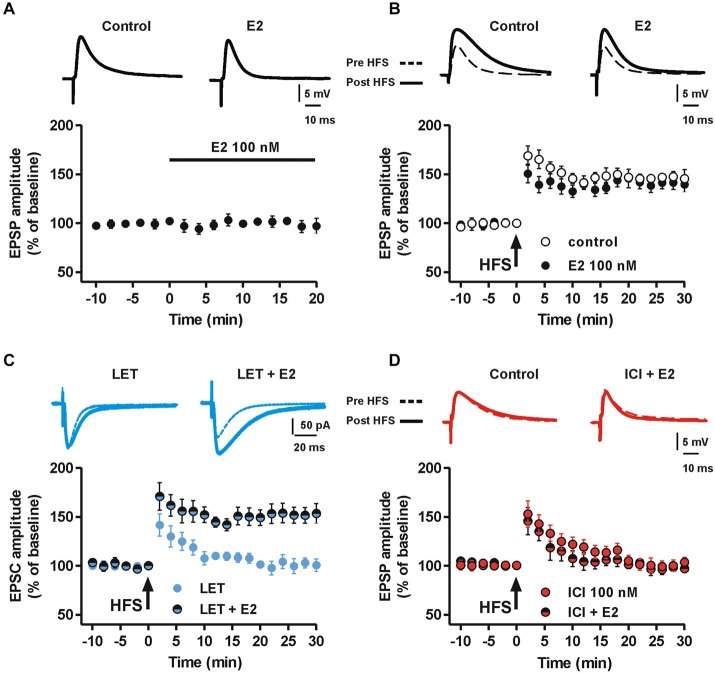
**Effect of E2 on synaptic transmission and the LTP in the presence of aromatase inhibition and ER blockade. (A)** Time-course of the EPSP amplitudes recorded from a group of MSNs before and after the application of 100 nM E2 for 20 min. Upper traces show EPSPs recorded from a MSN in control condition and 20 min after E2 application. **(B)** Time-course graph showing the LTP induced by HFS protocol in control conditions and in the presence of 100 nM E2. Upper pairs of traces showing EPSPs measured before and 30 min after the delivery of HFS protocol to induce LTP. **(C)** Time-course of the EPSC amplitude acquired before and after the application of the HFS protocol from a group of MSNs in the presence of 100 nM LET or LET plus 100 nM E2. Upper superimposed traces showing the EPSC traces acquired from MSNs before and 30 min after the HFS protocol. Note that E2 is able to induce LTP in the presence of ARO inhibition. **(D)** Time-course of the EPSP amplitude of MSNs recorded in the presence of 100 nM ICI or ICI plus 100 nM E2. Upper traces showing EPSP pairs recorded before and 30 min after delivery of the HFS protocol. Note the lack of effect of E2 in restoring the LTP in the presence of ICI.

To confirm the dependence of MSN LTP by E2, we first tested whether exogenous application of E2 affected both MSN glutamatergic synaptic transmission and LTP. MSNs recorded in the presence of 100 nM E2 for 20 min showed an EPSP that was not significantly different from control (*n* = 8, Figure 3A). Moreover, HFS delivered 15 min following E2 application produced a LTP not significantly different from control since the EPSP amplitude, measured 30 min after HFS in the presence of E2 was 148 ± 8.8% (*n* = 6) (Figure 3B). We subsequently analyzed whether E2 application was able to restore the LTP that was abolished in the presence of LET or ICI. MSNs recorded in the presence of LET co-applied with E2, showed a LTP similar to control. In fact, the EPSC amplitude, measured 30 min after the HFS, was increased by 153 ± 9.9% (*n* = 8, Figure 3C). Conversely, the co-application of ICI with E2 induced no LTP (*n* = 6, Figure 3D).

Taken together these data confirm the specificity of LET and ICI in the inhibition of E2 local synthesis and ER antagonism, respectively. Furthermore, they demonstrate that, while exogenous application of E2 is not able to influence basal synaptic transmission and the LTP of MSNs, the locally synthetized E2 is required for LTP induction.

### Inhibition of MSN LTP by Aromatase Blockade is Prevented by D1 but not D2 Dopamine Receptor Activation

**Figure 4:**
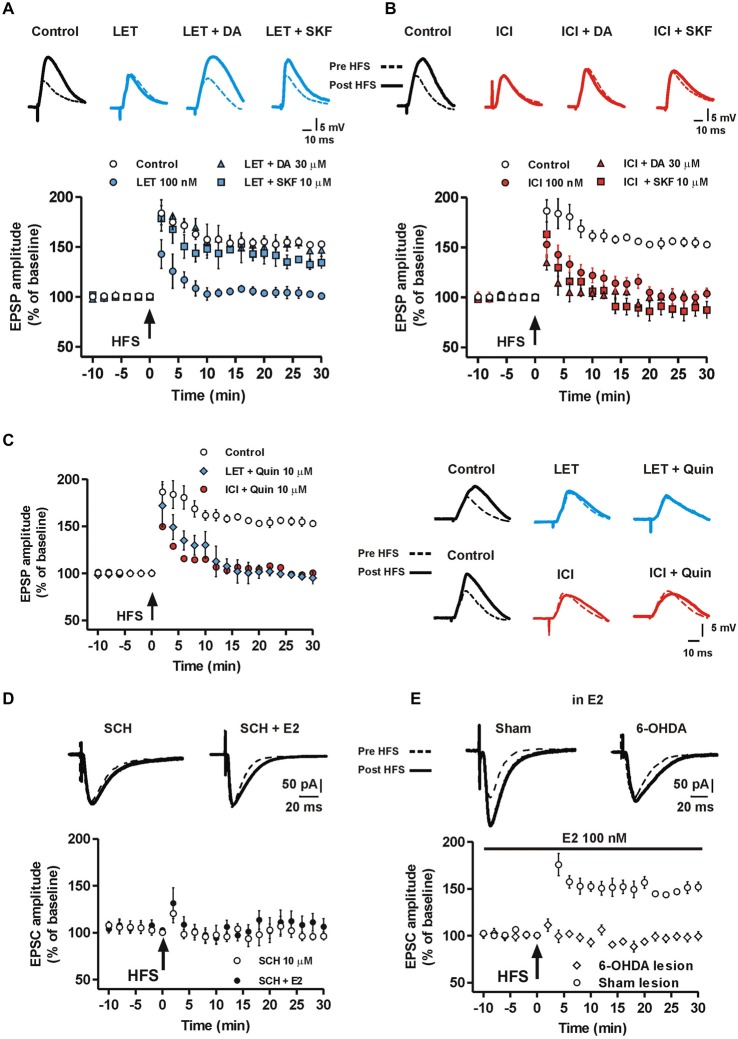
**Effect of DA and DA-R stimulation on MSN LTP in the presence of aromatase inhibition and ER antagonism. (A)** MSNs EPSP pairs of traces acquired before and after HFS and time-courses of the HFS effect on EPSP amplitude recorded in control conditions, in the presence of LET, LET plus 30 μM DA and LET plus 10 μM SKF. **(B)** EPSP traces and time-courses acquired in control conditions, in the presence of ICI, ICI plus 30 μM DA and ICI plus 10 μM SKF. **(C)** Time courses on the left show the effect of HFS on EPSP amplitude in control conditions, in the presence of LET plus 10 μM quinpirole and in the presence of ICI plus 10 μM quinpirole. EPSP traces on the right are recorded from MSNs before and after the HFS, upper traces: in control conditions, in the presence of LET or LET plus 10 μM quinpirole (LET + Quin); lower traces: in control conditions, in the presence of ICI or ICI plus quinpirole (ICI + Quin). Note that quinpirole in not able to restore LTP both in the presence of LET and ICI. **(D)** Time-course showing the EPSC amplitude of MSNs recorded in the presence of 10 μM of the D1R antagonist SCH or SCH plus 100 nM E2 co-applied before and following the HFS protocol. Upper EPSC traces recorded in MSNs before and 30 min after the HFS protocol in the presence of SCH or SCH plus E2. **(E)** Time-course graph of the EPSC amplitude measured in MSNs from 6-OHDA DA-denervated rats and sham-operated animals in the presence of 100 nM E2. Upper traces show EPSC acquired from a MSN of a 6-OHDA-lesioned rat and a sham-operated animal before and after the HFS protocol.

Since DA-R activation is critical for striatal LTP (Calabresi et al., 2007) and DA may affect ERs (Power et al., 1991; Olesen et al., 2005), it is likely that DA is able to overcome the block of E2 synthesis in preventing the induction of MSN LTP. Therefore, we verified the possible interaction between E2 and DA in controlling MSN LTP by testing whether administration of 30 μM DA could restore LTP in the presence of LET. In these conditions we found that all MSNs showed a robust LTP (*n* = 5) that was not significantly different from the one induced in the control condition (LET + DA, *n* = 5 vs. Control, *n* = 10, ANOVA, *F*_(20,210)_ = 0.12, *P* = 1.00, Figure 4A). Since DA crucially affects the induction of striatal LTP by binding the D1-like receptor (D1R) (Calabresi et al., 1992, 2007; Kerr and Wickens, 2001), we analyzed whether D1R activation was also able to restore LTP in the presence of LET and also tested a possible involvement of the D2-like DA receptor (D2R). Therefore, we induced LTP in the presence of LET plus either the D1R agonist SKF or the D2R agonist quinpirole. Similarly to the effect of DA, in the presence of LET plus 10 μM SKF, the LTP was induced in all the recorded neurons (*n* = 5) and was comparable to the one observed in control conditions (LET + SKF, *n* = 5 vs. Control, *n* = 10, ANOVA, *F*_(20,231)_ = 0.82, *P* = 0.69) and to that obtained in the presence of LET plus DA. Conversely, LTP prevented by LET was not rescued by 10 μM quinpirole (*n* = 5, Figure 4C). These findings suggest that exogenous DA, through the activation of D1R, is able to trigger events that overcome the block of E2 synthesis. In addition, they suggest that DA and E2 signaling may lead to activation of a common cascade of intracellular events crucial for LTP.

### Inhibition of LTP in MSNs by ER Antagonism is not Prevented by D1 or D2 Receptor Activation

Since the block of ERs, like ARO inhibition, prevented the MSN LTP, we verified the possibility that DA and D1R activation restored the LTP in the presence of ER antagonism. We found that either 30 μM DA or 10 μM SKF failed to re-establish the LTP in the presence of ICI (ICI + DA, *n* = 6, ICI + SKF, *n* = 6, Figure 4B). Similarly, the co-application of ICI with 10 μM quinpirole was not able to restore LTP (ICI + Quinpirole, *n* = 5, Figure 4C). Since DA overcame the block of endogenous E2 synthesis, but not that of ERs, interaction between D1R and ER in mediating the enzymatic cascade leading to LTP should be hypothesized.

### Effect of Exogenous Application of E2 on LTP of MSNs in the Presence of D1R Antagonism and in DA-Denervated Rats

Since LTP of MSNs is dependent on the activation of the D1 DA receptor, we next explored whether E2 application was able to induce LTP of MSNs in the presence of the D1 DA receptor antagonist SCH 23390 in control rats. Thus, we recorded MSNs after the application of E2 plus 10 μM SCH 23390 and we found that in these experimental conditions, LTP could not be induced (*n* = 5), similarly to what observed in the presence of SCH 23390 alone (*n* = 5) (Figure 4D). Finally, we tested whether E2 was able to restore LTP in the absence of endogenous striatal DA. To achieve this goal we unilaterally injected a group of rats with 6-OHDA into the MFB to produce striatal DA denervation and absence of both LTP and LTD in MSNs (Picconi et al., 2003; Calabresi et al., 2007). Thus, we recorded MSNs from slices of 6-OHDA-treated rats and sham-operated animals, after the application of 100 nM E2 in the attempt to overcome the absence of striatal DA. We found that in this conditions the HFS protocol failed to induce LTP in MSNs from 6-OHDA rats (*n* = 5, Figure 4E) but LTP could be induced in sham-lesioned animals (*n* = 5), providing a further confirmation that E2 is not sufficient to overcome the role of DA in the induction of LTP in MSNs.

### Activation of the cAMP-PKA Intracellular Pathway is Involved in the E2-Dependent LTP of MSNs

**Figure 5:**
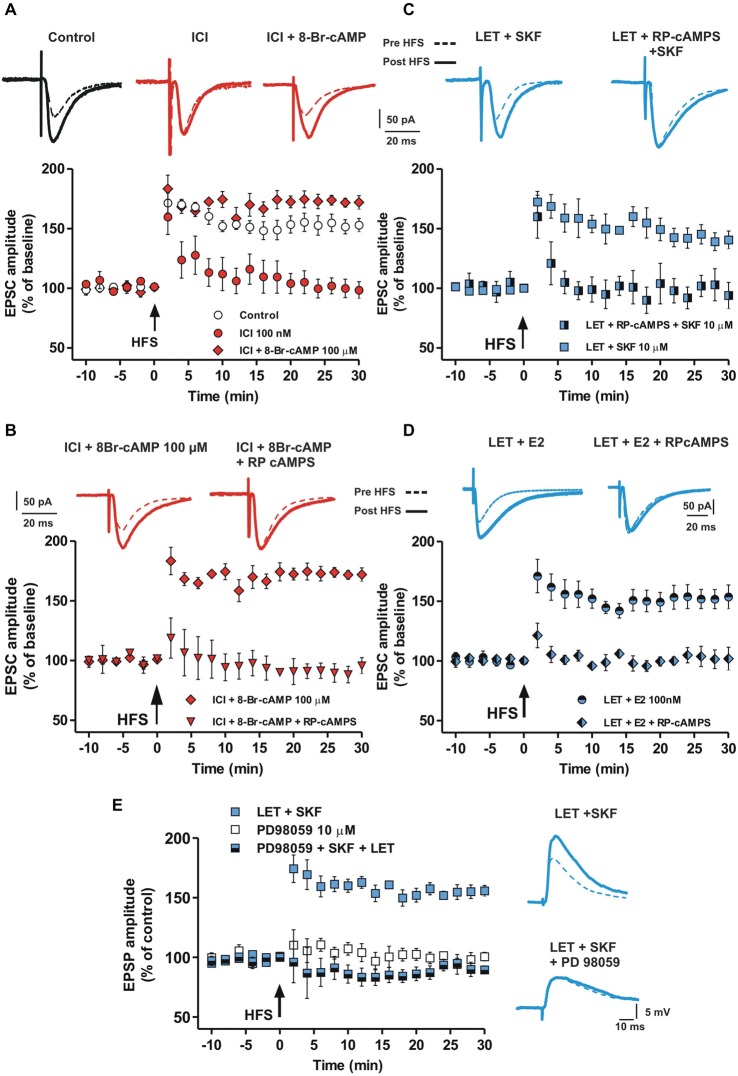
**Involvement of cAMP-PKA pathway in the D1R-dependent MSN LTP in the presence of aromatase inhibition and ER antagonism. (A)** EPSC traces recorded from MSNs before and after the HFS in control conditions and in the presence of ICI and ICI plus 100 μM 8-Br-cAMP. Graph shows the time-course of the HFS effect on the amplitude of EPSC recorded in control conditions, in the presence of ICI and ICI plus 8-Br-cAMP. **(B)** EPSC traces and time-course graph of HFS effect in the presence of ICI plus 8-Br-cAMP and ICI plus 8-Br-cAMP plus 100 μM RP-cAMPS. **(C)** EPSC pairs of traces and time-course of HFS effect on EPSC amplitudes acquired in the presence of LET plus SKF and LET plus RP-cAMPS plus SKF. **(D)** EPSC pairs and time-course of the effect of 100 nM E2 applied in the presence of 100 nM LET or in the presence of LET plus 100 μM RPcAMPS. **(E)** EPSP pairs and time-course of HFS effect on EPSP amplitudes recorded in the presence of LET plus SKF and LET plus SKF plus 10 μM PD98059.

The cAMP-PKA intracellular pathway is activated by DA in the induction of LTP in MSNs (Calabresi et al., 2007). Since MSN LTP is prevented by the ER blockade, we verified whether this LTP could be restored by enhancing the cAMP intracellular level. Thus, MSNs were injected with 100 μM of the cAMP analog 8-Br-cAMP and EPSCs were recorded in the presence of ICI. In these conditions we found a robust LTP (Control vs. ICI + 8-Br-cAMP, *n* = 5, *F*_(20,168)_ = 1.49, *P* = 0.09, Figure 5A), while LTP was always prevented by using the standard intracellular solution (ICI, *n* = 6). This finding suggests that the effect of ER blockade in preventing LTP may depend on reduced levels of cAMP. Accordingly, 100 μM of the PKA inhibitor RP-cAMPS prevented the LTP restoration by the cAMP analog in the presence of ICI (ICI + 8-Br-cAMP + RP-cAMPS, *n* = 4, Figure 5B), suggesting that ER stimulation concurs to LTP induction through the activation of the cAMP-PKA pathway.

To further confirm the involvement of the cAMP-PKA intracellular enzymatic pathway, we recorded MSNs injected with the PKA inhibitor RP-cAMPS in the presence of both LET and SKF. In these condition, SKF was not able to restore a normal LTP in the presence of LET (LET + SKF + RP-cAMPS, *n* = 6, Figure 5C). Notably, RPcAMPS prevented the rescue of MSN LTP by E2 in the presence of LET (*n* = 5, Figure 5D), suggesting that both D1R and ER activation may mediate the LTP in MSNs *via* stimulation of the cAMP-PKA intracellular pathway.

### LTP Induction in the Striatal MSNs Depends on ERK Activation

E2 can facilitate synaptic plasticity also by ER dependent activation of the extracellular signal-regulated kinase (ERK) pathway in different cerebral regions (Bi et al., 2000; Dominguez et al., 2009) and ERK is involved in striatal LTP (Cerovic et al., 2015). Since we found that D1R activation restores LTP in the absence of endogenous E2, but does not restore it in the presence of ER blockade, we explored whether the D1R- and ER-dependent MSN LTP could also be mediated by ERK activation. To test for this possibility we evaluated the effect of the ERK1/2 inhibition by PD98059 in an experimental condition in which the lack of LTP produced by inhibition of E2 synthesis was prevented by the D1R agonist SKF. In the presence of LET plus 10 μM PD98059, SKF was not able to restore LTP (LET + SKF + PD98059, *n* = 5), mimicking the effect of bath application of 10 μM PD98059 alone (*n* = 4) (Figure 5E). Taken together these findings suggest that ERs, activated by E2 or D1R stimulation, mediate the induction of MSN LTP involving the cAMP-PKA and the ERK intracellular enzymatic cascades.

### Activation of ERs and D1Rs is Involved in the Induction of LTP in Striatal Cholinergic Interneurons

We found that DA was able to restore LTP in the presence of LET through D1R activation in all the tested neurons. Since some striatal MSNs do not express D1Rs, we hypothesized a role for striatal cholinergic interneurons (ChIs) that express D1-like receptors (Aosaki et al., 1998) and impinge on MSNs (Calabresi et al., 2006; Tozzi et al., 2011) in mediating the D1R-dependent restoration of LTP in MSNs not expressing D1Rs (putative D2R-expressing MSNs).

Since ERs were shown to be expressed in both MSNs and ChIs (Almey et al., 2012), suggesting that E2 effect on MSNs might also be mediated by striatal ChIs projections, we tested whether inhibition of E2 synthesis by LET could interfere with synaptic transmission and plasticity at this level.

**Figure 6:**
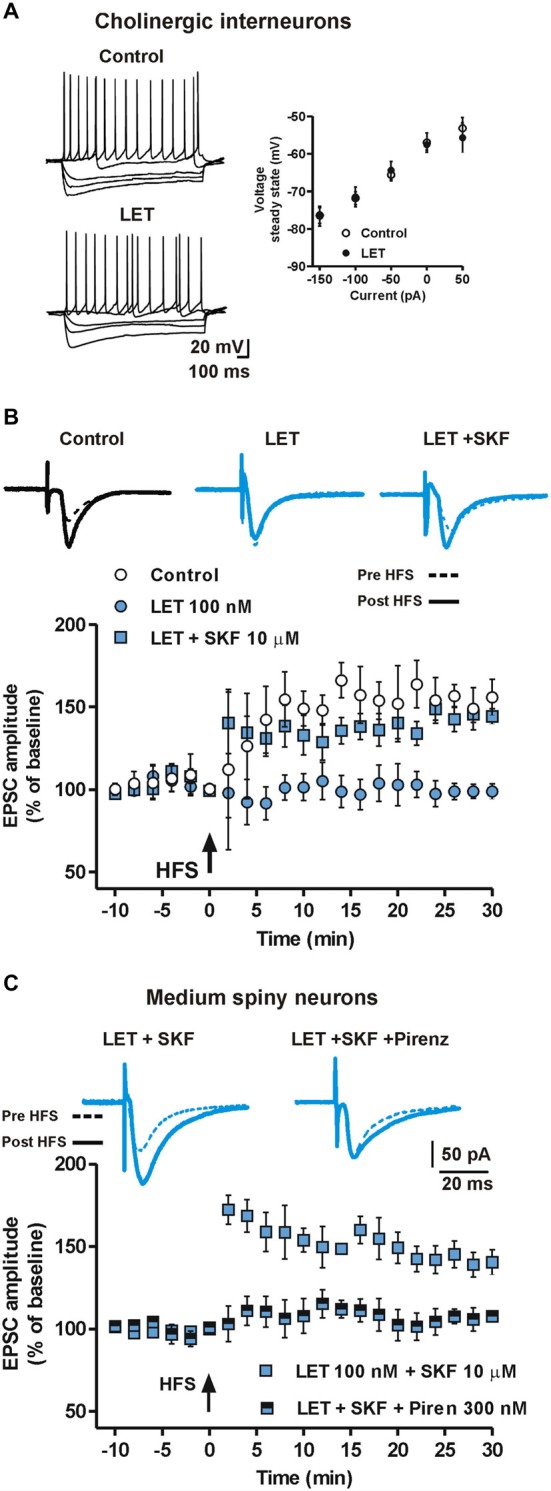
**Endogenous E2 formation is involved in LTP of ChIs and mediates LTP in MSNs by stimulating cholinergic M1 receptor. (A)** Representative voltage traces (left) and current-voltage plot, measured at the steady state (right), of ChIs during the injection of hyperpolarizing and depolarizing steps of currents in control conditions and in the presence of LET. **(B)** EPSC pairs and time-course of EPSC amplitudes recorded from ChIs before and after HFS in control conditions, in the presence of LET and LET plus SKF.** (C)** EPSC pairs of traces and time-courses of EPSC amplitudes recorded before and after HFS from MSNs in the presence of LET plus SKF and LET plus SKF plus 300 nM pirenzepine.

We found that while bath application of 100 nM LET did not alter *per se* the firing pattern discharge, the current-voltage relationship (*n* = 5, Figure 6A) and the EPSC amplitude of ChIs (*n* = 4, data not shown), it completely prevented LTP (*n* = 5, Figure 6B) revealing a pivotal role of endogenous E2 in the induction of synaptic potentiation also in striatal ChIs. However, we found that in the presence of LET plus SKF, the LTP recorded from ChIs could be restored (*n* = 7) and that it was not significantly different from the one obtained in control condition (LET + SKF, *n* = 7 vs. control, *n* = 5, ANOVA, *F*_(20,210)_ = 1.14, *P* = 0.31, Figure 6B).

#### E2 and D1R Stimulation Modulate Cholinergic tone onto Striatal MSNs and Affect MSN LTP

Released acetylcholine (Ach) by striatal ChIs directly modulates MSNs function by binding pre- and post-synaptic cholinergic receptors. M1 muscarinic receptors are particularly abundant at MSNs postsynaptic sites and mediate multiple biochemical cascades pivotal for the regulation of thalamo-cortical inputs and the modulation of DA signaling to MSNs. Specifically, M1R stimulation, beside its effects on the IP3-mediated intracellular pathway, can also increase cAMP intracellular levels (Sánchez-Lemus and Arias-Montaño, 2006). Thus, E2 may influence LTP in MSNs by controlling cAMP levels either directly *via* ERs expressed by MSNs, and indirectly by ERs located on ChIs. Therefore, we verified the effect of M1R blockade, with 300 nM pirenzepine, on the capability of the D1R agonist SKF to recover the loss of LTP observed in MSNs in the presence of LET. In this condition, the LTP was prevented (*n* = 8, Figure 6C) suggesting that M1R activation could significantly contribute to the LTP induction in MSNs. Taken together these data point out a pivotal role of ChIs, Ach release and M1R stimulation in the E2 dependent LTP induction in MSNs.

## Discussion

In the present study we demonstrate for the first time to our knowledge that the locally synthesized E2 is crucial for the induction of the LTP in the striatum by interacting with the downstream enzymatic cascade activated by DA.

Using electrophysiological recordings from single striatal neurons, we report that E2 synthesis and ER activation are required for the induction of LTP in both MSNs and ChIs, but not for its maintenance. In fact, LTP was completely abolished in the presence of inhibition of the E2 synthesizing enzyme ARO with LET or the blocking agent for ER (ICI), both administered a few minutes before HFS. Moreover, we found that neither LTD nor depotentiation of LTP in MSNs was affected by LET and ICI.

The effect of the locally synthesized E2 on the LTP induction appears to be specifically related to synaptic plasticity events rather than to an action on membrane excitability or basal synaptic transmission. Accordingly, agents able to interfere with E2 synthesis (LET) or with its binding to ERs (ICI), did not modify *per se* both the membrane electrical properties and basal synaptic response of the striatal neurons.

Moreover, the exogenous administered E2 did not affect the baseline activity of striatal neurons. Instead, it was able to restore the LTP in the presence of ARO inhibition, suggesting a specific role of E2 in LTP. We also found that exogenous E2 was not able to further enhance the LTP amplitude indicating that the endogenous E2 is sufficient to permit a full HFS-dependent LTP.

The crucial role of the locally-synthesized E2 for the induction of striatal LTP is in line with previous evidence showing the lack of LTP in the vestibular nuclei (Grassi et al., 2009, 2010, 2011; Tanaka and Sokabe, 2012; Pettorossi et al., 2013; Scarduzio et al., 2013) of male rat when LET or ICI was delivered few minutes before LTP induction. Unlike these immediate effects on LTP, a chronic influence of prolonged LET treatments on synaptic plasticity has been demonstrated in the hippocampus of female mice where blockade of E2 synthesis produced a spine synapse loss and impairment of LTP (Vierk et al., 2012, 2014).

Although our study shows that locally synthesized E2 is crucial for the induction of the LTP in the striatum, whether suppression of E2 synthesis by LET reduces a tonically synthesized E2 or prevents a phasic enhancement of E2 occurring during HFS is still unknown. Both hypotheses are plausible. A tonic influence of E2 has been demonstrated by prolonged block of ARO that reduces the amount of E2 in the medium of slice cultures (Vierk et al., 2012). However, there is also evidence for an E2 synthesis which is dependent on phasic activity, as shown by the activation of ARO following Ca^2+^ entry at pre- and post-synaptic level as a consequence of the NMDA receptor activation (Kimoto et al., 2001; Balthazart and Ball, 2006; Hojo et al., 2008). Since, in our experiments, the ARO inhibitor was administered just few minutes before the LTP induction, we suggest that the induction of LTP is more likely dependent on the phasic synthesis of E2 than the tonic one. Therefore, we hypothesize that the intense stimulation of glutamatergic fibers, during the LTP-inducing protocol, produces an enhancement of Ca^2+^ level at pre- and/or post-synaptic sites that, in turn, activates ARO for increasing the synthesis of E2 at both pre-synaptic and/or post-synaptic level. Moreover, also glial cells might be involved in the synthesis of E2.

Beside the functional demonstration for the involvement of local synthesis of E2 in striatal LTP, there is immunohistochemical evidence that ARO and ERs are expressed in the striatum of male and female mice, as well as in female rats (Küppers and Beyer, 1998, 1999; Almey et al., 2012). E2 may affect neuronal signaling in the dorsal striatum via binding ERα, ERβ and GPER-1 (Almey et al., 2012). In our experiments we found that the ERα antagonist ICI-182780 was able to fully prevent LTP induction of striatal MSNs.

It should be noted that in our study we investigated the influence of endogenous E2 on striatal plasticity only in the male rat, in spite of most evidences for the E2 involvement in the striatum that were provided in the female (Hampson and Kimura, 1988; Becker, 1990a,b; Mermelstein et al., 1996; Boulware et al., 2005; Olesen et al., 2005). The reason for using male rats was that a consistent effect of endogenous E2 on the LTP has been previously shown in male rats (Grassi et al., 2009, 2010, 2011; Tanaka and Sokabe, 2012; Pettorossi et al., 2013; Scarduzio et al., 2013), while it was more variable in the female, depending on the level of circulating E2 during the estrous cycle (Pettorossi et al., 2011; Grassi et al., 2012). In the present study we observed a clear and robust striatal E2-dependent LTP in the male. Although it is possible that a similar phenomenon might occur also in the striatum of female rat, as suggested by previous behavioral studies (Hampson and Kimura, 1988; Becker, 1990a,b; Mermelstein et al., 1996; Boulware et al., 2005; Olesen et al., 2005), possible difference of gender sensitivities of striatal neurons to E2 might also occur. It is possible, in fact, that different expression of nuclear and membrane ERs in female vs. males might cause gender differences in the interaction of E2 with DA.

However, it appears evident from our study that the expression of ARO and ERs, already shown in the male, are sufficient for allowing the endogenous E2 to play an immediate effect on the induction of striatal LTP.

The evidence that LTP in striatal MSNs and ChIs depends on the local synthesis of E2 suggested us to verify the possible interaction with DA, since the dopaminergic input is also crucial for LTP (Calabresi et al., 2007). In fact, the LTP was restored in all MSNs when either exogenous DA or the D1R agonist SKF was applied under block of E2 synthesis. This reversal did not occur with the application of the D2R agonist quinpirole. Conversely, E2 was not able to rescue LTP in a model in which we prevented the release of DA by striatal DA denervation with injection of 6-OHDA into the MFB (Picconi et al., 2003). Therefore, these results demonstrate that the D1R activation may overcome the lack of endogenous E2 triggering the downstream enzymatic cascade for LTP induction while administration of E2 does not overcome the lack of DA, suggesting a crucial permissive action of DA for inducing the LTP.

The experiments performed with the receptor antagonists confirm the strict interplay between DA and E2. As expected, the E2-dependent LTP was fully prevented by the block of D1R (SCH 23390). Conversely, the finding that the D1R agonist was not able to restore the LTP in the presence of ER block (ICI) was surprising and it is not in line with that observed under ARO blockade. Since DA cannot overcome the E2 action downstream the receptor activation, we can hypothesize a cross-talk between D1R and ER or the activation of ER by DA, as previously reported (Power et al., 1991; Walters et al., 2002).

ICI had been also reported to activate GPER-1 (Langer et al., 2010) that, in turn, is able to stimulate cAMP production, Ca^2+^ release from intracellular stores and to modulate ERK/MAPK (Filardo, 2002). However, the hypothesis that the activation of GPER-1 by ICI could trigger inhibitory pathways for neuronal excitability cannot be excluded.

Thus, since we found that DA and SKF failed to restore the LTP in the presence of ICI, we might speculate that, in parallel with the antagonism of ERα/β, a possible stimulation of GPER-1 by ICI could oppose the action of DA or SKF in the activation of the cAMP/PKA pathway and LTP restoration of MSNs. Future studies should be performed for an in-deep analysis of the specific contribution of ERα, ERβ and GPER-1 signaling pathways on striatal LTP.

Concerning the events downstream the E2 receptor activation, we found that E2-mediated LTP in striatal MSNs requires necessarily the DA-mediated activation of the cAMP-PKA intracellular pathway (Calabresi et al., 2007). In fact, the antagonist of PKA (RP-cAMPS) prevented the induction of LTP by either SKF or E2 in the presence of LET. This suggests that E2 and DA converge on the cAMP-PKA intracellular pathway to facilitate LTP and the contemporary activation of D1R and ER leads to the level of cAMP necessary for LTP induction. The possibility that E2 modulates MSNs LTP involving presynaptic mechanisms of action must be considered. Our experiments using intracellular modulators of the cAMP-PKA pathway are not conclusive for a pre- vs. post-synaptic site of action. In fact, we cannot exclude that the intracellularly applied compounds used to modulate cAMP and PKA at postsynaptic sites might partially diffuse to also influence presynaptic terminals. Moreover, the activation of ERs located on dopaminergic terminals may increase the release of DA (Zheng, 2009) and facilitate the induction of LTP.

Thus, it is possible that D1R activation by SKF is able to restore LTP in the presence of LET leading to a level of cAMP similar to that normally obtained by the combined HFS-dependent activation of ER and D1R. Therefore, the different rescue capability of DA and E2 in inducing LTP may be explained by considering that the level of cAMP induced by DA could be higher than that obtained by E2.

Our results also suggest that the ERK intracellular pathway is involved in the enzymatic cascade downstream the activation of ER and D1R, since the ERK inhibitor PD98059 prevented the effect of SKF in restoring, in MSNs, the LTP in the presence of LET. Taken together, these findings are in agreement with previous reports showing different rapid effects of E2 in brain structures including the control of Ca^2+^ influx (Wu et al., 2005; Zhao et al., 2005), activation of cAMP (Gu and Moss, 1996) c-Src (Nethrapalli et al., 2001), αCaMKII cascade (Sawai et al., 2002), ERK1/2 and CREB phosphorylation (Szego et al., 2006) and the modulation of neuronal excitability through MAP kinase-induced calpain activation (Zadran et al., 2009).

Since the D1R agonist SKF, but not the D2R agonist quinpirole, was able to restore LTP in MSNs, we expected that some of these neurons, namely those not expressing D1Rs (putative D2R-expressing MSNs), should be unresponsive to application of DA or D1R agonist. Surprisingly, we found that D1R stimulation restores the LTP in all the recorded MSNs in the presence of inhibition of E2 synthesis.

Given the heterogeneity of expression of D1- and D2-like DA receptors localized in striatal MSNs (Calabresi et al., 2014), we hypothesized that the recorded MSNs reflected this distribution. However, the possibility that we randomly recorded only D1R-expressing MSNs cannot be excluded. Future experiments allowing unequivocal identification of the recorded MSN should be performed. We also hypothesized a role for ChIs, since these interneurons express both D1-like (mainly D5) receptors (Aosaki et al., 1998) and ERs (Almey et al., 2012) and they can modulate the activity of the entire MSN population by M1R stimulation (Tozzi et al., 2011) and by M4R stimulation in a subpopulation of MSNs co-expressing D1Rs (Surmeier et al., 2014). M1R activation is known to stimulate the enhancement of intracellular Ca^2+^ levels via the activation of the G_q/11_/PLC/IP_3_ intracellular pathway; however, it has also been shown to mediate cAMP increase in striatal MSNs (Sánchez-Lemus and Arias-Montaño, 2006). Accordingly, we demonstrated that also the LTP of ChIs depends on endogenous E2 and that DA, through the activation of a D1-like receptor, is able to restore the LTP of this interneuron when its induction is prevented by LET. Furthermore, we used the M1R antagonist pirenzepine to prevent the effects of ChIs on MSNs and we found that this drug blocked in MSNs the induction of LTP in the presence of D1R agonism and ARO inhibition (Figure 6).

Moreover, since the activation of M1R has been shown to mediate cAMP increase in striatal MSNs (Sánchez-Lemus and Arias-Montaño, 2006), we hypothesize that ChIs could also play a role in the E2 and DA dependent LTP of MSNs by enhancing in these neurons cAMP levels via M1R. On the other hand, the effect of cholinergic modulation of MSN activity may result in a more complex regulation of the cAMP levels in D1R-expressing MSNs that also co-express M1 and M4 muscarinic receptors (Sánchez-Lemus and Arias-Montaño, 2006; Surmeier et al., 2014).

In fact, muscarinic receptors located on MSNs can potentially enhance or diminish cAMP levels by acting on M1 and/or M4 receptors respectively (Sánchez-Lemus and Arias-Montaño, 2006; Surmeier et al., 2014). However, since the different subtypes of muscarinic receptors appear not to be uniformly distributed to the entire MSN population (M4Rs co-localizing on D1Rs-expressing MSNs, M1Rs being more ubiquitous to all MSNs) a same unique final effect of acetylcholine (Ach) involving M4Rs in all MSNs is not conceivable. It is also plausible that the final effect of Ach release in the modulation of MSN activity is also dependent on the strength of synaptic inputs impinging on ChIs, thus different stimulation patterns might produce different effects. Nevertheless, a possible influence of cholinergic projections arising from peduncolopontine neurons converging to striatal microcircuits (Dautan et al., 2014) might contribute to the modulatory effects of Ach on MSNs LTP.

Overall, our findings show that LTP in MSNs strictly depends on the presence of DA and the locally synthesized E2. LTP can be induced when increment of corticostriatal inputs (Stoetzner et al., 2010) and a tonic or dynamic release of DA occur (Schultz, 2007; Lerner and Kreitzer, 2011).

Our study also reveals the importance of events triggered by excitation of striatal glutamatergic pathway interacting with the DA signaling in the LTP induction of MSNs. Endogenous E2, in fact, probably synthesized during the high frequency activation of corticostriatal pathway is needed for the LTP induction through ER activation, facilitating post-synaptic mechanisms leading to LTP and also regulating the DA release (Becker, 1990a,b; Watson et al., 2006). Furthermore, our data suggest that DA can activate ERs probably by a close interaction between D1Rs and ERs and that this co-activation is possibly required in order to drive intracellular signals able to trigger LTP. Thus, we suggest that ER stimulation represents a pivotal step for the long-term modulation of synaptic changes in both striatal MSNs and ChIs.

The possibility that E2 and other locally synthesized neurosteroids can participate, together with DA, to trigger striatal synaptic plasticity is particularly intriguing in the view that motor skill acquisition is a phenomenon dependent on the ability of striatal neurons to undergo long-term changes of synaptic plasticity (Calabresi et al., 1992, 2007; Suzuki et al., 2001; Wickens et al., 2003; Lerner and Kreitzer, 2011) requiring combined activation of the corticostriatal and the nigro-striatal pathways. Furthermore, the role of E2 and neurosteroids in modulating synaptic plasticity in pathological conditions may be of particular interest, especially for those pathologies involving the nucleus striatum such as Parkinson’s disease.

## Author and Contributors

AT, AdI, CG, MT, VD, AQ-V, MDM, PM, CC, MDF performed and analyzed the experiments; AT, SG, VP and PC designed the experiments and wrote the manuscript. All authors provided important intellectual content and critically revised the final version of the manuscript.

## Conflict of Interest Statement

Paolo Calabresi is a member of the editorial boards of Lancet Neurology, Journal of Neuroscience, Movement Disorders, and Synapse and receives research support from Biogen, Lundbeck, Merck-Serono, Sanofi-Aventis, UCB, Fondazione Santa Lucia IRCCS and Ministero della Salute. All other authors reported no biomedical financial interests or potential conflicts of interest.
